# Antiepileptic drugs and foetal disorders: analysis of 20-year data from the pharmacovigilance center

**DOI:** 10.3389/fphar.2025.1556598

**Published:** 2025-02-26

**Authors:** Zejun Ji, Jianjun Nie, Qingli Shen, Zhonghua Fu

**Affiliations:** ^1^ Department of Clinical Pharmacy, The First Affiliated Hospital of Nanyang Medical College, Nanyang, Henan, China; ^2^ Department of Pharmacy, Henan Provincial People’s Hospital, People’s Hospital of Zhengzhou University, School of Clinical Medicine, Henan University, Zhengzhou, Henan, China

**Keywords:** antiepileptic drug, foetal disorder, foetal heart rhythm disorders, foetal growth restriction, valproic acid

## Abstract

**Background:**

For women of childbearing age, the risks of uncontrolled epilepsy to the mother and fetus need to be balanced against the potential teratogenic effects of antiepileptic drugs (AEDs). The combined use of different types of AEDs has become a potential treatment option for the effective control of epileptic symptoms, while different studies present significant difference between the combined use of AEDs and foetal toxicity, which need a large comprehensive study to clarify the relation.

**Objective:**

The study aims to analyze data from the U.S. Food and Drug Administration (FDA) Adverse Event Reporting System (FAERS) to explore the impact of monotherapy or polytherapy of AEDs on foetal and infant disorders.

**Methods:**

Bayesian analysis and non-proportional methods were employed to assess the association between AED use and foetal disorders based on the FAERS database from the first quarter of 2004 to the fourth quarter of 2023. The clinical characteristics and outcome of patients were also investigated.

**Results:**

The study identified significant correlation between foetal disorders and the first and second generation AEDs, with RORs of 3.8 and 4.9, respectively. Valproic acid monotherapy showed the highest correlation with foetal disorders (ROR = 15.8, PRR = 16.3, IC025 = 3.8) and was uniquely associated with male reproductive toxicity. The risk of foetal disorders associated with combination therapies varied depending on the specific AEDs combination, with some increasing and others decreasing the risk compared to monotherapy.

**Conclusion:**

The analysis of the reports from FAERS database identified correlation between foetal disorders and AEDs and provided a comprehensive overview of the incidence and prognosis of different AEDs monotherapy and combination, which may provide some advice for the selection of drug for women of childbearing age.

## 1 Introduction

Epilepsy is a common nervous system disease, which affects about 1% of the world’s population, and the prevalence rate of women of childbearing age is about 0.3%–0.7% (Thigpen and Owens, 2022). For women of childbearing age, the risks of uncontrolled epilepsy to the mother and fetus need to be balanced against the potential teratogenic effects of antiepileptic drugs (AEDs) (Perucca et al., 2024), thus the selection of AEDs, either as monotherapy or in combination is a focus of clinical research. Studies have shown that different AEDs are associated with specific disease risks in offspring (Tomson et al., 2019; Asranna et al., 2018). For example, valproic acid may increase the risk of neural tube defects, cardiac abnormalities, urogenital malformations and skeletal deformities (Tomson et al., 2016); carbamazepine, phenytoin and phenobarbital may be associated with a higher risk of neural tube defects, cardiovascular defects and oral clefts (Hernández-Díaz et al., 2000); topiramate shows a higher association with oral cleft and hypospadias. While some newer AEDs show a trend of reduced foetal toxicity (Reimers and Brodtkorb, 2012). The different teratogenicity of AEDs may significantly influence the medication choice for women of childbearing age.

During pregnancy, due to changes in the physiological environment of pregnant women, the blood concentration of AEDs may significantly decrease (Pennell et al., 2022; Arfman et al., 2020; Pennell et al., 2022; Arfman et al., 2020), often requiring an increase in dosage to maintain effective control of epilepsy. However, the teratogenic potential of some AEDs shows a strong correlation with increased dosage (Hernández-Díaz et al., 2000), which may add complexity to the treatment. Besides, the combined use of different types of AEDs has become a potential treatment option for the effective control of epileptic symptoms. It is worth noting that different studies present significant difference between the combined use of AEDs and foetal toxicity (Vajda et al., 2019), which need a large comprehensive study to clarify the relation. Therefore, the pharmacovigilance study aims to analyze data from the U.S. Food and Drug Administration (FDA) Adverse Event Reporting System (FAERS) to explore the impact of monotherapy or polytherapy from different generations of AEDs on foetal and infant disorders, and to compare the effects of two AEDs used in combination versus monotherapy on foetal and infant disorders. The study may provide a comprehensive overview of foetal disorders associated with AEDs, serving as a supplement of previous studies which only include limited outcomes such as growth restriction, congenital malformations, and death.

## 2 Methods

### 2.1 Data source

The FAERS is a publicly accessible database maintained by the US FDA, which contains data voluntarily reported by individuals involved in drug use. OpenVigil 2.1 is an open-source pharmacovigilance data extraction, mining, and analysis tool specifically designed for use with the FAERS database. It is important to note that OpenVigil 2.1 operates exclusively on preprocessed FAERS data, which has been filtered to remove most duplicates and reports with incomplete information. Some fetuses have not yet been born, and related reports may be filed under the identity of the mother.

In the FAERS database, adverse events (AEs) are classified using preferred terms (PTs) from the Medical Dictionary for Regulatory Activities (MedDRA) (version 25.0). A specific PT may be linked with multiple higher-level terms (HLTs), higher-level group terms (HLGTs), and system organ classes (SOCs). Additionally, PTs that indicate symptoms, signs, investigations, or diagnoses of potential significance are organized into Standardized MedDRA Queries (SMQs) to portray specific medical conditions. The research concentrated on the specific SMQ concerning foetal disorders, including 118 PTs (Supplementary Table S1).

### 2.2 Data extraction

In this study, data from the FAERS database of AEDs between the first quarter of 2004 and the fourth quarter of 2023 was collected and retrospectively analyzed. Among the AEDs, the first generation of drugs are phenytoin, phenobarbital, valproic acid, and carbamazepine, the second generation drugs include lamotrigine, oxcarbazepine, topiramate and evetiracetam, while the third generation drugs comprise zonisamide and lacosamide. Duplicate entries with the same PSR number were removed prior to analysis. The narrow SMQ and PT dimensions were used to analyze the correlation between foetal disorders and AEDs, also the correlation of the combined application of two AEDs with foetal disorders was analyzed.

### 2.3 Data mining

A disproportionality analysis was performed using a case/non-case methodology. The Reporting Odds Ratio (ROR) (Sakaeda et al., 2013) and Proportional Reporting Ratio (PRR) (Slattery et al., 2013), were calculated to identify potential reporting disproportionality signals in foetal disorder related to AEDs. An exploratory disproportionality approach comparing foetal disorder related to AEDs versus all other drugs (non-cases) reported in the FAERS database was conducted. The study also paid close attention to the AEs of foetal disorders with a positive ROR (lower limit of the 95% CI > 1 with at least 3 cases) especially those were not documented in the FDA label. The Bayesian Information Component (IC) (Hauben and Bate, 2009), which is considerd more accurate for the analysis of the small sample size of reports, was calculated to decrease the risk of detecting false signals. The equations and criteria for the three algorithms are shown in Supplementary Table S2.

### 2.4 Statistical analysis

All data were statistically analyzed using IBM^®^ SPSS^®^ Statistics (version 26), and Sankey plot were used to demonstrate the clinical characteristics of cases with AEDs-related foetal disorders. Pearson chi-square test or Fisher exact test was used to compare the congenital malformation rate and mortality rate among different AEDs. p-value less than 0.05 was considered statistically significant. All graphs were plotted online with the help of chiplot.

## 3 Results

### 3.1 Descriptive analysis

**FIGURE 1:**
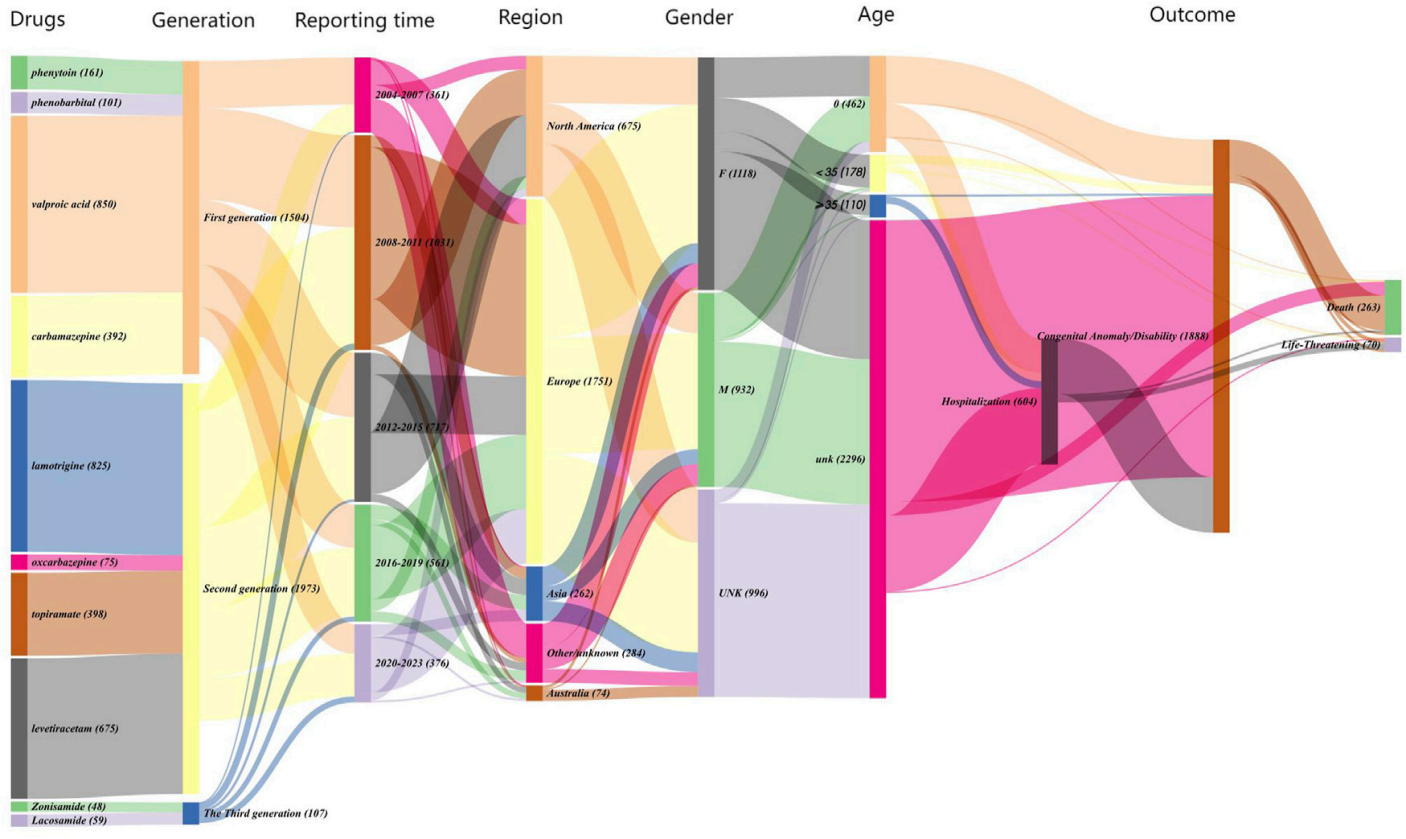
Sankey plot of clinical characteristics of patients with AED-related foetal disorders.

**FIGURE 2:**
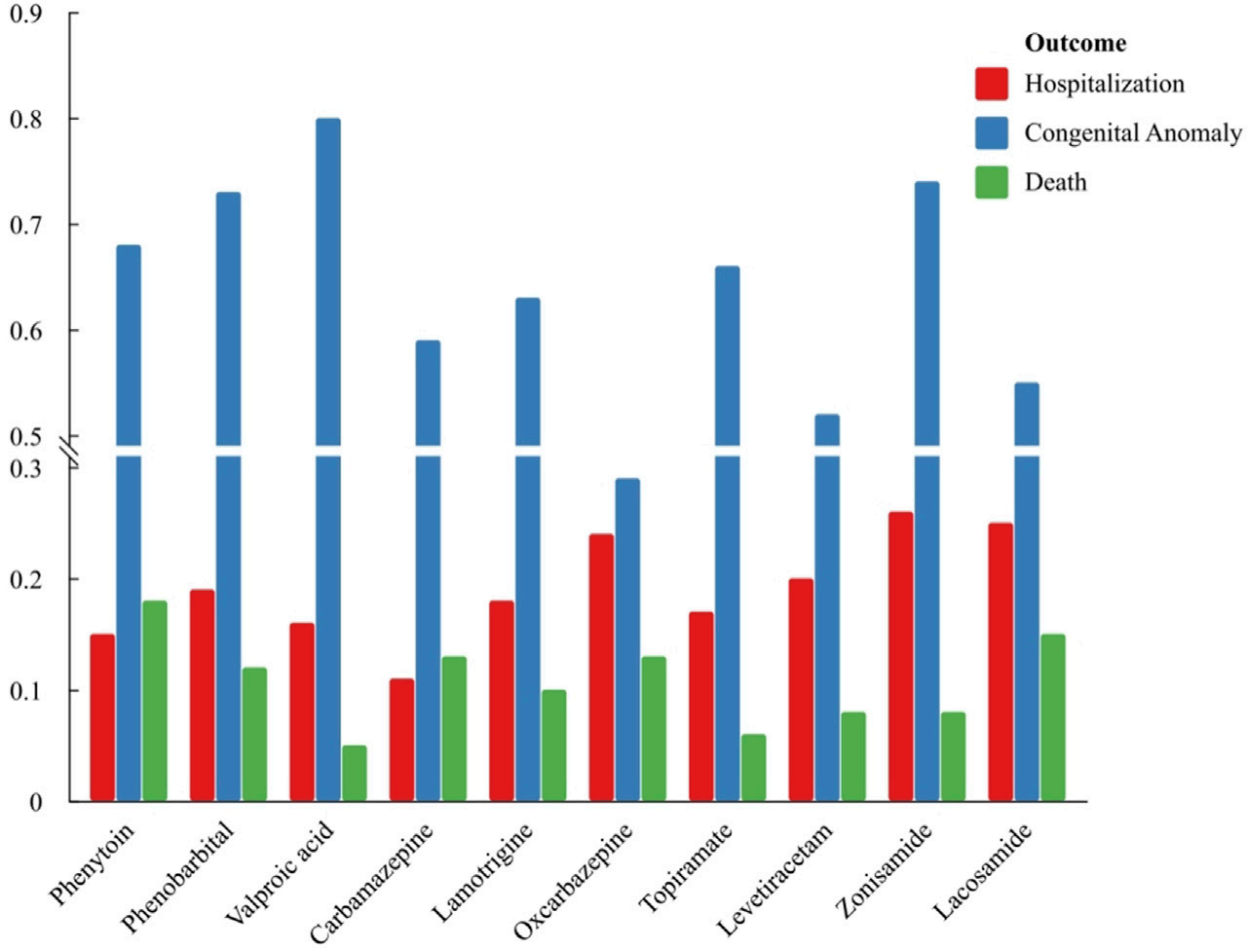
The outcomes of reports related to AEDs and foetal diseases.

A total of 3,046 reports of foetal disorders associated with AEDs were included in this study, of which the number of reports associated with second-generation AEDs was highest, accounting for more than 50%, showing in Figures 1, 2. Reports of foetal diseases associated with the third-generation AEDs remained relatively rare. However, there had been an increasing reports of the third-generation AEDs since 2012, which may be related to the perceived greater safety and subsequently widespread clinical use of third-generation AEDs. Among the reported cases of foetal disorders related to AEDs, Europe accounted for a relatively high proportion, more than 50%, and a relatively high proportion (>30%) was reported between 2008 and 2011. removing those with unknown age in the reported cases, the reporting proportion of infants was higher than that of adults, and the proportion of boys among infants was higher than that of girls, while 93.1% of adults were females. foetal disorders related to AEDs are often accompanied by adverse outcomes such as prolonged hospital stays, congenital anomalies/disabilities and death. Among them, congenital anomalies accounted for a relatively high proportion of 62.0%, and more than 10% of fetuses may be life-threatening or die. The third-generation AEDs showed significantly higher rates of foetal hospitalization and mortality than the first and second generation AEDs.

### 3.2 Bayesian and nonproportional analyses

According to the positive criteria, the first and second genenration AEDs all indicated a strong correlation between the AEs and the drugs use, when comparing the group “Monotherapy + Polytherapy” and group “Monotherapy”, there was a statistically significant difference between the two groups for the first and second genenration AEDs, as shown in Table 1. However, in the “Monotherapy” group, there was no positive correlation between foetal disorders and the third generation AEDs. As for the specific drugs, there was no significant statistical significance between foetal disorders and phenytoin, zonisamide, or lacosamide. Valproic acid in group “Monotherapy” showed the highest correlation with foetal disorders (ROR = 15.8, PRR = 16.3, IC025 = 3.8).

**TABLE 1:** Signal strength of AEDs related foetal disorders.

Drug	Monotherapy				Monotherapy + polytherapy			
	N	PRR (χ2)	ROR (95%two-sided CI)	IC(IC025)	N	PRR (χ2)	ROR (95%two-sided CI)	IC(IC025)
First generation	**801**	**3.8(1601.7)**	**3.8(3.5–4.1)**	**2.9(2.8)**	**1211**	**8.5(7672.1)**	**8.6(8.2–9.2)**	**3.0(2.9)**
Phenytoin	39	1.5 (5.4)	1.4 (1.1–2.0)	0.5 (0.0^a^)	149	3.8 (304.4)	3.8 (3.3–4.5)	1.9 (1.6)
Phenobarbital	15	5.9 (56.7)	5.9 (3.6–9.8)	2.4 (1.5)	77	8.2 (478.8)	8.3 (6.6–10.4)	3.0 (2.6)
Valproic acid	599	15.8 (8131)	16.3 (15.0–17.7)	3.9 (3.8)	796	14.4 (9593.4)	14.7 (13.7–15.8)	3.8 (3.7)
Carbamazepine	151	4.0 (345)	4.0 (3.4–4.7)	2 (1.7)	336	6.2 (1428.8)	6.2 (5.6–6.9)	2.6 (2.4)
Second generation	**905**	**4.9(2682.8)**	**4.9(4.6–5.2)**	**2.2(2.1)**	**1531**	**6.1(6167.8)**	**6.2(5.9–6.5)**	**2.5(2.5)**
Lamotrigine	393	5.0 (1237.6)	5.0 (4.6–5.6)	2.3 (2.1)	729	7.0 (3631.3)	7.1 (6.6–7.6)	2.8 (2.6)
Oxcarbazepine	24	1.6 (4.7)	1.6 (1.1–2.4)	0.7 (0)	63	2.8 (68.7)	2.8 (2.2–3.5)	1.4 (1.0)
Topiramate	189	4.5 (513.3)	4.6 (3.9–5.3)	2.2 (1.9)	376	6.6 (1770.6)	6.7 (6.1–7.4)	2.7 (2.5)
Levetiracetam	299	5.5 (1090.1)	5.6 (5.0–6.2)	2.4 (2.2)	643	6.7 (3016.6)	6.7 (6.2–7.3)	2.6 (2.5)
Third generation	**21**	**1.1(0.3)**	**1.1(0.7–1.8)**	**0.1(-0.6)**	**92**	**2.3(65.6)**	**2.3(1.9–2.8)**	**1.1(0.8)**
Zonisamide	8	1.9 (2.7)	1.9 (1.0^b^-3.9)	0.8 (-0.4)	39	4.3 (97.0)	4.4 (3.2–6.0)	2.0 (1.5)
Lacosamide	13	0.9 (0)	0.9 (0.5–1.6)	−0.2 (-1.1)	55	1.7 (15.7)	1.7 (1.2–2.2)	0.7 (0.3)

^a^ Real value greater than 0.

^b^ Real value less than 1. Bold: sum of data.

**FIGURE 3:**
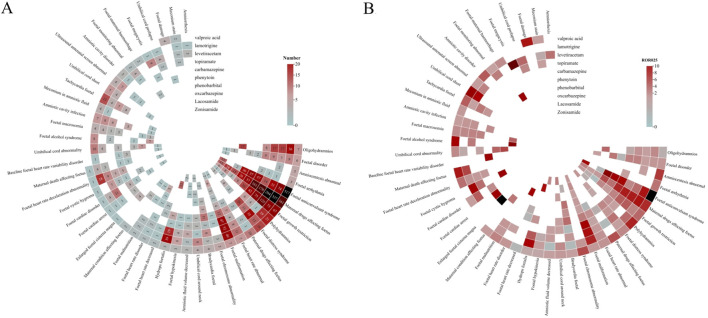
Correlation heatmap of AEDs-related foetal disorders **(A)**. The number of reports of AEDS-related fetal disorders. **(B)** ROR025 value of AEDS-related fetal disorders.

The analysis of AEDs with PTs including in the narrow SMQ of foetal disorders was also counducted. 3,567 AEs were reported for 10 AEDs, as shown in Figure 3. Among them, the most frequently reported PTs were: maternal drugs affecting foetus (1,261), foetal anticonvulsant syndrome (797), foetal growth restriction (424), foetal malformation (184), oligohydramnios (102), and foetal distress syndrome (100). All of the ten AEDs contained the PTs of maternal drugs affecting foetus, foetal growth restriction, and foetal malformation. For the PTs of foetal anticonvulsant syndrome, valproic acid, phenobarbital, phenytoin, zonisamide, carbamazepine, lamotrigine, levetiracetam, and topiramate showed statistically significant correlation, besides, valproic acid accounted for 64.9% of the total AEs, and the correlation was the highest (n = 517, ROR = 264.5). A total of 11 cases of foetal megacystis were reported, which showed a strong correlation with levetiracetam (n = 9, ROR = 83.4). Paternal drugs affecting foetus was reported in 15 cases, which was related to the use of valproic acid, carbamazepine, levetiracetam, and lamotrigine, while only valproic acid showed positive correlation (n = 10, ROR = 6.8). Umbilical cord shortening showed a strong correlation with lamotrigine (n = 7, ROR = 30.6). Enlarged foetal cisterna magna showed a strong correlation with carbamazepine (n = 4, ROR = 30.6). The third generation drug lacosamide showed strong correlation with foetal alcohol syndrome (n = 7, ROR = 46.4), hydrops foetalis (n = 13, ROR = 11.8), and foetal malformation (n = 6, ROR = 2.3).

### 3.3 Correlation between two AEDs combined and foetal disorders

**FIGURE 4:**
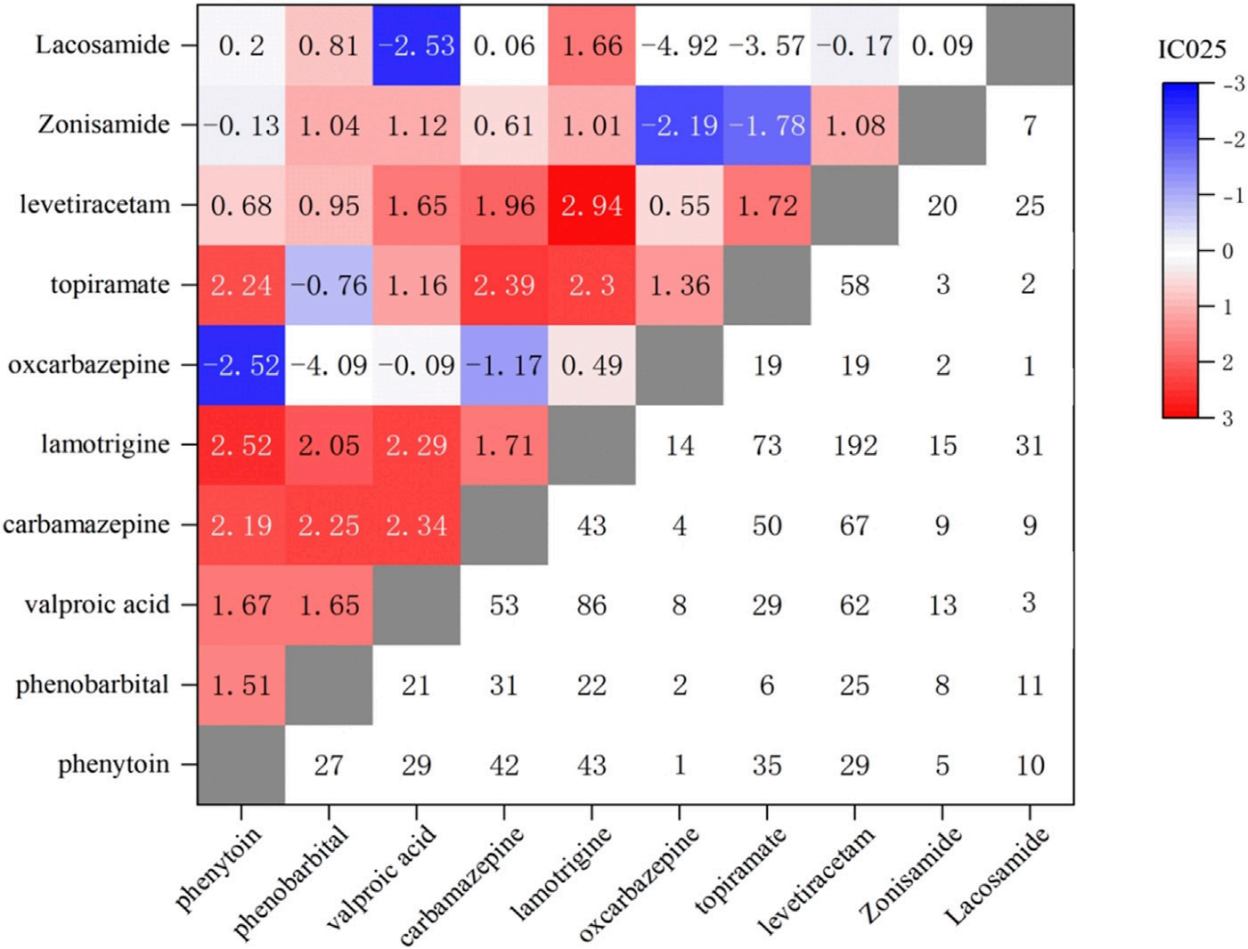
Correlation heatmap of foetal disease caused by combined treatment with two AEDs. Lower right: Number of foetal disorders reports of two AEDs; Upper left: IC025 values of foetal disorders of two AEDs.

In the study, the number of reported cases involving the combination use of two AEDs was 1,264, and the correlation between foetal disorders and combination use was analyzed, as shown in Figure 4 and Table 2. The most commonly reported combination of the two AEDs were lamotrigine and levetiracetam (n = 192), lamotrigine and valproic acid (n = 86), and lamotrigine and topiramate (n = 73), followed by levetiracetam and carbamazepine (n = 67). Lamotrigine combined with other AEDs was more frequently reported (519 cases, 41.1%). The combination use of lamotrigine and levetiracetam showed a highest correlation with foetal disorders, with ROR values higher than the monotherapy of each (10.0 vs. 5.0, 10.0 vs. 5.6). The combination use of lamotrigine with phenytoin/phenobarbital/topiramate/carbamazepine/lacosamide showed a higher association with foetal disorders compared to the single-drug use. There were 304 cases of valproic acid combination use with other AEDs, while the ROR values for these combinations were lower than those observed in valproic acid monotherapy. Additionally, some combinations also showed lower ROR values compared to the individual drugs, including oxcarbazepine with phenytoin/phenobarbital, topiramate with phenobarbital/valproic acid, levetiracetam with phenobarbital/valproic acid, zonisamide with oxcarbazepine/topiramate, and lacosamide with valproic acid/oxcarbazepine/topiramate. Interestingly, zonisamide and lacosamide showed no statistical association with foetal disorders when used as monotherapy, while used in combination, they demonstrated a significant statistical association.

**TABLE 2:** ROR and PRR values for fetal diseases associated with the combined use of two antiepileptic drugs.

Drugs	phenytoin	Phenobarbital	valproic acid	carbamazepine	Lamotrigine	Oxcarbazepine	Topiramate	Levetiracetam	Zonisamide	Lacosamide
Phenytoin		5.0 (82.5)	5.5 (103.1)	7.4 (224.6)*	9.4 (312.7)*	1.1 (0)^†^	8.1 (210.3)*	2.7 (28.4)	3.4 (6.3)*	2.7 (9.5)*
Phenobarbital	5.0 (3.4–7.4)		6.1 (84.7)	8.4 (196.2)*	8.3 (133.3)*	0.8 (0)^†^	1.7 (1.2)^†^	3.4 (39.8)^†^	6.6 (32.7)*	4.3 (24.4)
Valproic acid	5.6 (3.9–8.0)	6.2 (4.0–9.5)		7.8 (307.2)	6.8 (421.9)	2.5 (5.7)	3.8 (57.0)^†^	4.6 (168.9)^†^	5.0 (37.8)	0.7 (0.1)^†^
Carbamazepine	7.5 (5.5–10.1)	8.6 (6.0–12.2)	7.9 (6–10.4)		5.1 (138.5)*	1.7 (0.6)	8.2 (309.0)*	5.6 (249.1)*	4.0 (18.0)	2.6 (7.4)
Lamotrigine	9.5 (7.0–12.9)	8.4 (5.5–12.8)	6.9 (5.6–8.6)	5.2 (3.8–7.0)		2.9 (16)	7 (375.8)*	9.8 (1503.3)*	4.3 (34.5)	5.4 (106.1)*
Oxcarbazepine	1.1 (0.3–4.5)	0.8 (0.1–5.5)	2.5 (1.2–5.0)	1.7 (0.6–4.5.0)	2.9 (1.7–5.0)		5.1 (58.3)*	2.7 (19.3)	1.5 (0)^†^	0.4 (0.5)^†^
Topiramate	8.2 (5.9–11.4)	1.7 (0.8–3.9)	3.8 (2.6–5.5)	8.3 (6.3–11.0)	7.2 (5.7–9.0)	5.1 (3.3–8.1)		4.9 (173.4)	1.3 (0)^†^	0.5 (0.7)^†^
Levetiracetam	2.7 (1.9–3.8)	3.4 (2.3–5.0)	4.6 (3.6–5.9)	5.7 (4.4–7.2)	10 (8.7–11.5)	2.7 (1.8–4.3)	4.9 (3.8–6.3)		4.0 (42.3)	1.5 (3.7)
Zonisamide	3.4 (1.4–8.3)	6.7 (3.3–13.4)	5 (2.9–8.7.0)	4.1 (2.1–7.9)	4.3 (2.6–7.2)	1.5 (0.4–6.1)	1.3 (0.4–4.2)	4 (2.6–6.3)		3.1 (8.2)*
Lacosamide	2.8 (1.5–5.1)	4.3 (2.4–7.8)	0.7 (0.2–2.3)	2.6 (1.4–5.0)	5.4 (3.8–7.7)	0.4 (0.1–2.7)	0.5 (0.1–1.9)	1.5 (1.0–2.2)	3.2 (1.5–6.6)	

Lower left: ROR, value (95% confidence interval); Upper right: PRR, value (χ2); *: Higher than the monotherapy of either drug; †: Lower than the monotherapy of either drug.

## 4 Discussion

The objective of the study was to analyze the correlation between the use of AEDs and foetal disorders, utilizing these data to understand the clinical characteristics and difference among various drug interventions. To our knowledge, this is the first study to retrospectively analyze the impact of monotherapy or polytherapy with antiepileptic drugs on foetal disorders from a pharmacovigilance perspective leveraging the FAERS database.

The analysis indicates that the correlation between the different combined use of two AEDs and foetal disorders is not consistent, primarily depending on the specific AEDs combined. Compared to monotherapy, the combined use of certain AEDs may increase the correlation with foetal disorders, but it is challenging to accurately identify which drugs contribute most to adverse outcomes. Valproic acid showed a particularly strong association with foetal disorders in both monotherapy and combination use, previous studies have confirmed its association with a high incidence of adverse foetal outcomes in reproductive populations (Ornoy et al., 2023). While valproic acid demonstrated a higher ROR for monotherapy with foetal disorders compared with combined use. Previous studies have observed that the incidence of congenital malformations in offspring is higher when valproic acid is used in combination with lamotrigine, carbamazepine, and phenobarbital than with monotherapy of these drugs (Holmes et al., 2011; Matalon et al., 2002; Zaccara and Perucca, 2014), confirming that valproic acid is a major factor influencing foetal disorders when used in combination (Ornoy et al., 2023; Morrow et al., 2006; Ornoy et al., 2023; Morrow et al., 2006). The use of specific combinations of AEDs, such as phenytoin with oxcarbazepine, valproate with topiramate, phenobarbital with levetiracetam, and lacosamide with topiramate, can significantly reduce the correlation with foetal disorders compared to the monotherapy of these AEDs. The phenomenon may be attributed to the reduced dosage of individual AEDs when used in combination, thereby decreasing the risk of foetal disorders (Tomson et al., 2015; Verrotti et al., 2015) (Tomson et al., 2015; Verrotti et al., 2015), as previous studies have found that the reproductive toxicity of AEDs such as valproate, phenytoin, phenobarbital, and lacosamide is dose-dependent, with increased reproductive toxicity risks at higher doses (Tomson et al., 2019; Mete et al., 2016; Tomson et al., 2019; Mete et al., 2016). It may be possible to reduce the potential risks to the fetus while ensuring therapeutic efficacy through the rational use of AED combinations.

Lamotrigine and levetiracetam also demonstrated a high reporting frequency, which may be related to their superior efficacy, tolerability, and fewer side effects compared to the first-generation AEDs, leading to their prioritized recommendation in guidelines for populations with reproductive needs (Pack et al., 2024; Singh and Verma, 2019). An interesting finding contrary to previous studies is that lamotrigine and levetiracetam also showed a high correlation with foetal disorders, which may be related to their frequent co-administration with other AEDs, an article shows comedication altered the clearance of lamotrigine to the greatest extent ±70% because it is affected by both enzyme inducers and inhibitors (Johannessen Landmark et al., 2012). A total of 797 cases related to foetal anticonvulsant syndrome were reported, with 65% related to valproic acid, making it the most common AED causing the syndrome (Ornoy et al., 2023). Phenytoin and carbamazepine both showed high ROR associated with the syndrome, previous literature also indicates that phenytoin and carbamazepine are high-risk factors for foetal anticonvulsant syndrome and should be avoided during pregnancy (Moore et al., 2000; Clayton-Smith et al., 2019). Foetal growth restriction is one of the most commonly used indicators to assess the toxicity of AEDs in offspring exposed during pregnancy (Viale et al., 2015)^.^ This study showed that all AEDs were reported causing foetal growth restriction, particularly for topiramate, which showed a significant correlation with (Christensen et al., 2024; Hernández-Díaz et al., 2018). Lamotrigine, levetiracetam, and carbamazepine were reported with higher frequencies of foetal cardiac rhythm abnormalities. Carbamazepine showed a higher correlation with foetal cardiac arrest, phenytoin was associated with baseline foetal heart rate variability disorder, and oxcarbazepine was related to foetal heart rate disorder. This may be due to these AEDs causing embryonic hypoxia, subsequent reoxygenation, and reactive oxygen species production, leading to tissue damage and induction of embryonic arrhythmias (Etemad et al., 2012). It has also been found that AEDs use by males can cause foetal disorders, but only with valproic acid, previous literature demonstrates that valproic acid affects male fertility, while no such association is found with levetiracetam or lamotrigine, or oxcarbazepine, thus males with reproductive needs should avoid using valproic acid (Markoula et al., 2020; Asghar et al., 2024). The study also found that in some reports, other drugs were used together, such as levothyroxine, folic acid, sedative hypnotics and antidepressants. Although these medications were not the focus of the current investigation, their potential impact on foetal disorders will be a focus of future research.

This study capitalizes on the analysis of real-world clinical data and employs data mining techniques, however, it is accompanied by several inherent limitations. Firstly, our methodology could not reliably differentiate between accurate and erroneous or inaccurate reported data, which may introduce bias. Secondly, although we extracted statistics from basic patient information, the precision of comorbidities and medication history remained uncertain, potentially introducing confounding factors and uncertainties into our analysis. Besides, for the limited information of the database, it is hard to acquire the accurate dosage and biological level of the drug. Thirdly, data mining utilizing Bayesian and proportional hazard analysis can only ascertain statistical association, not causality between AEs and drug administration. Despite the limitations, our analysis indicates that the use of two AEDs in combination often shows a stronger correlation with foetal disorders compared to monotherapy, although the outcomes vary significantly with different AED combination. However, due to the lack of in-depth research on the mechanisms of action of AED combinations on foetal disorders, establishing a clear causal relationship between the combination use of these drugs and adverse outcomes remains challenging. Therefore, it is necessary to conduct large-scale clinical trials to more comprehensively delineate these relationships.

## Data Availability

The original contributions presented in the study are included in the article/Supplementary Material, further inquiries can be directed to the corresponding author.
