# Supplementary material for: Antiepileptic drugs and foetal disorders: analysis of 20-year data from the pharmacovigilance center
Source: Front Pharmacol. 2025 Feb 26;16:1556598. doi: 10.3389/fphar.2025.1556598 (PMC11897480; doi:10.3389/fphar.2025.1556598)
Supplement: Supplementary file 1 [file Table1.docx]

Table S1. The PT term and code included in the narrow SMQ of foetal disorders.

| PT | Code | PT | Code |
| --- | --- | --- | --- |
| Alpha 1 foetoprotein amniotic fluid abnormal | 10001776 | Foetal malnutrition | 10016862 |
| Alpha 1 foetoprotein amniotic fluid decreased | 10060746 | Foetal megacystis | 10073660 |
| Alpha 1 foetoprotein amniotic fluid increased | 10001778 | Foetal methotrexate syndrome | 10071183 |
| Amniocentesis abnormal | 10001959 | Foetal monitoring abnormal | 10071507 |
| Amniorrhexis | 10051641 | Foetal movement disorder | 10077576 |
| Amniorrhoea | 10066470 | Foetal musculoskeletal imaging abnormal | 10077580 |
| Amnioscopy abnormal | 10001965 | Foetal non-stress test abnormal | 10071516 |
| Amniotic cavity disorder | 10060936 | Foetal renal imaging abnormal | 10077581 |
| Amniotic cavity infection | 10060937 | Foetal renal impairment | 10078987 |
| Amniotic fluid erythropoietin level increased | 10064845 | Foetal retinoid syndrome | 10073720 |
| Amniotic fluid index abnormal | 10067079 | Foetal surgery | 10080563 |
| Amniotic fluid volume decreased | 10063356 | Foetal tachyarrhythmia | 10077575 |
| Amniotic fluid volume increased | 10063357 | Foetal therapeutic procedure | 10057294 |
| Amniotic infection syndrome of Blane | 10051407 | Foetal tobacco syndrome | 10086945 |
| Anaesthetic complication foetal | 10052850 | Foetal vascular malperfusion | 10085689 |
| Angiotensin converting enzyme inhibitor foetopathy | 10051098 | Foetal warfarin syndrome | 10051445 |
| Baseline foetal heart rate variability disorder | 10074638 | Foetal-maternal haemorrhage | 10016871 |
| Biopsy chorionic villous abnormal | 10048537 | Gestational age test abnormal | 10072479 |
| Biopsy foetal abnormal | 10058370 | Haemorrhage foetal | 10061191 |
| Bradycardia foetal | 10006094 | Hydrops foetalis | 10020529 |
| Cerebral haemorrhage foetal | 10050157 | Hypocalvaria | 10073670 |
| Cerebral infarction foetal | 10008119 | Hypopituitarism foetal | 10021068 |
| Chronic villitis of unknown etiology | 10072271 | Increased foetal movements | 10086915 |
| Cordocentesis | 10066447 | Intrauterine infection | 10056254 |
| Diabetic foetopathy | 10059116 | Maternal condition affecting foetus | 10057673 |
| Diethylstilboestrol syndrome | 10012780 | Maternal death affecting foetus | 10026912 |
| Discordant twin | 10069150 | Maternal drugs affecting foetus | 10026923 |
| Ductus arteriosus stenosis foetal | 10013808 | Maternal hypertension affecting foetus | 10026924 |
| Enlarged foetal cisterna magna | 10081422 | Meconium in amniotic fluid | 10051133 |
| Erythroblastosis foetalis | 10015251 | Meconium increased | 10027059 |
| Foetal acidosis | 10050347 | Meconium peritonitis | 10058113 |
| Foetal alcohol syndrome | 10016845 | Meconium stain | 10057028 |
| Foetal anaemia | 10077577 | Mirror syndrome | 10068875 |
| Foetal anticonvulsant syndrome | 10066485 | Nonreassuring foetal heart rate pattern | 10074641 |
| Foetal arrhythmia | 10016847 | Oligohydramnios | 10030289 |
| Foetal biophysical profile score abnormal | 10078124 | Omphalorrhexis | 10064270 |
| Foetal biophysical profile score equivocal | 10078123 | Paternal drugs affecting foetus | 10050425 |
| Foetal cardiac arrest | 10084280 | Polyhydramnios | 10036079 |
| Foetal cardiac disorder | 10052088 | Prenatal screening test abnormal | 10069151 |
| Foetal cerebrovascular disorder | 10053601 | Radiation injury affecting foetus | 10053602 |
| Foetal chromosome abnormality | 10064041 | Sinusoidal foetal heart rate pattern | 10074643 |
| Foetal compartment fluid collection | 10077628 | Tachycardia foetal | 10043074 |
| Foetal cystic hygroma | 10052011 | Thalidomide embryopathy | 10071249 |
| Foetal damage | 10016852 | Twin reversed arterial perfusion sequence malformation | 10073455 |
| Foetal disorder | 10061157 | Ultrasound antenatal screen abnormal | 10045400 |
| Foetal distress syndrome | 10016855 | Ultrasound foetal abnormal | 10077578 |
| Foetal gastrointestinal tract imaging abnormal | 10077579 | Umbilical artery vascular resistance increased | 10084637 |
| Foetal growth abnormality | 10077582 | Umbilical cord abnormality | 10048596 |
| Foetal growth restriction | 10070531 | Umbilical cord around neck | 10045447 |
| Foetal heart rate abnormal | 10051139 | Umbilical cord compression | 10045451 |
| Foetal heart rate acceleration abnormality | 10074642 | Umbilical cord cyst | 10079122 |
| Foetal heart rate deceleration abnormality | 10074636 | Umbilical cord haemorrhage | 10064534 |
| Foetal heart rate decreased | 10051136 | Umbilical cord occlusion | 10076714 |
| Foetal heart rate disorder | 10061158 | Umbilical cord prolapse | 10045452 |
| Foetal heart rate increased | 10051138 | Umbilical cord short | 10045453 |
| Foetal heart rate indeterminate | 10079882 | Umbilical cord thrombosis | 10071652 |
| Foetal hypokinesia | 10068461 | Umbilical cord vascular disorder | 10045454 |
| Foetal macrosomia | 10053700 | Vesicoamniotic shunt | 10068236 |
| Foetal malformation | 10060919 | Virilism foetal | 10047487 |

Table S2. Summary of major algorithms applied for signal detection

| Algorithms | Equation^#^ | Criteria |
| --- | --- | --- |
| ROR | ROR=(a/b)/(c/d)  95%CI=e^ln(ROR)±1.96(1/a+1/b+1/c+1/d)^0.5^ | ROR(95% CI-low)>1, N≥3 |
| PRR | PRR=(a/(a+c))/(b/(b+d))  χ2 =∑[(O-E)2/E]，［O=a, E =(a+b)(a+c)/(a+b+c+d)］ | PRR≥2, χ2≥4,  N≥3 |
| BCPNN | IC=log_2_a(a+b+c+d)/((a+c)(a+b))  IC025=e^ln(IC)-1.96(1/a+1/b+1/c+1/d)^0.5^ | IC025>0 |

^#^ a: number of reports containing both the suspect drug and the suspect adverse drug reaction. b: number of reports containing the suspect adverse drug reaction with other medications (except the drug of interest). c: number of reports containing the suspect drug with other adverse drug reactions (except the event of interest). d: number of reports containing other medications and other adverse drug reactions.ROR, reporting odds ratio; CI, confidence interval; N, the number of co-occurrences; PRR, proportional reporting ratio; χ2, chi-squared; BCPNN, Bayesian confidence propagation neural network; IC, information component; IC025, the lower limit of the 95% two-sided CI of the IC;
